# Prevention of vaginal and rectal HIV transmission by antiretroviral combinations in humanized mice

**DOI:** 10.1371/journal.pone.0184303

**Published:** 2017-09-07

**Authors:** Philippe A. Gallay, Udayan Chatterji, Aaron Kirchhoff, Angel Gandarilla, Manjula Gunawardana, Richard B. Pyles, Mark A. Marzinke, John A. Moss, Marc M. Baum

**Affiliations:** 1 Department of Immunology & Microbiology, The Scripps Research Institute; La Jolla, California, United States of America; 2 Department of Chemistry, Oak Crest Institute of Science; Monrovia, California, United States of America; 3 Department of Pediatrics, University of Texas Medical Branch, Galveston, Texas, United States of America; 4 Department of Medicine, Johns Hopkins University, Baltimore, Maryland, United States of America; 5 Department of Pathology, Johns Hopkins University, Baltimore, Maryland, United States of America; George Mason University, UNITED STATES

## Abstract

With more than 7,000 new HIV infections daily worldwide, there is an urgent need for non-vaccine biomedical prevention (nBP) strategies that are safe, effective, and acceptable. Clinical trials have demonstrated that pre-exposure prophylaxis (PrEP) with antiretrovirals (ARVs) can be effective at preventing HIV infection. In contrast, other trials using the same ARVs failed to show consistent efficacy. Topical (vaginal and rectal) dosing is a promising regimen for HIV PrEP as it leads to low systematic drug exposure. A series of titration studies were carried out in bone marrow/liver/thymus (BLT) mice aimed at determining the adequate drug concentrations applied vaginally or rectally that offer protection against rectal or vaginal HIV challenge. The dose-response relationship of these agents was measured and showed that topical tenofovir disoproxil fumarate (TDF) and emtricitabine (FTC) can offer 100% protection against rectal or vaginal HIV challenges. From the challenge data, EC_50_ values of 4.6 μM for TDF and 0.6 μM for FTC for HIV vaginal administration and 6.1 μM TDF and 0.18 μM for FTC for rectal administration were obtained. These findings suggest that the BLT mouse model is highly suitable for studying the dose-response relationship in single and combination ARV studies of vaginal or rectal HIV exposure. Application of this sensitive HIV infection model to more complex binary and ternary ARV combinations, particularly where agents have different mechanisms of action, should allow selection of optimal ARV combinations to be advanced into pre-clinical and clinical development as nBP products.

## Introduction

More than 7,000 new HIV infections occur daily [1], creating an urgent need to identify new strategies that prevent transmission of the virus. Non-vaccine biomedical prevention (nBP) methods such as topical or systemic pre-exposure prophylaxis (PrEP) are promising strategies to stop the spread of HIV [2–15]. To date, multiple clinical trials based on tenofovir (TFV) dosing regimes, frequently in combination with emtricitabine (FTC), provided evidence that PrEP significantly reduced HIV infection in individuals [16–24]. The CAPRISA 004 trial provided the first demonstration that a topical microbicide could preclude HIV transmission in humans. Specifically, a 1% tenofovir (TVF) gel used pericoitally decreased the incidence of HIV transmission in South African women by 39% [16]. Two additional trials with 1% tenofovir gel with pericoital (VOICE) [25] and daily (FACTS) [26] dosing regimens failed to provide efficacy against new sexual HIV infections.

In the ASPIRE trial of a monthly intravaginal ring delivering dapivirine, incidence of HIV infection was significantly reduced for women who wore the ring consistently, but poor adherence in some participant groups, particularly among younger women, led to low overall efficacy (27% risk reduction) [27]. In these trials, poor adherence to the prophylaxis regimens is a primary factor in the lack of efficacy; however, additional factors may be responsible for disparate efficacy results, as demonstrated by a randomized pharmacokinetic crossover study [28] where the tissue concentration advantage (>100x) seen in gel dosing compared to oral dosing was not reflected in seroconversion outcomes of the CAPRISA and VOICE trials. This may indicate that factors beyond antiviral effect may reduce topical PrEP efficacy. Possible explanations include concentration dependent tissue toxicity from TFV, tenofovir-diphosphate (TFV-DP) effects, or dose-frequency dependence effects from the gel vehicle. Both may be below the sensitivity or beyond the scope of safety evaluation. Nevertheless, the conflicting trial results serve to highlight the complexity of interactions between HIV and the host at the mucosal surfaces during virus acquisition and further demonstrate an urgent need for suitable *in vivo* model systems that can elucidate the mechanisms responsible for such contradictory results.

The analysis of the efficacy of selected microbicidal candidates requires animal models. The macaque model, currently used for vaginal HIV transmission studies, involves infection with either simian immunodeficiency virus (SIV) or SIV/HIV (SHIV) chimeric viruses [29–34]. This model presents some limitations: it does not support HIV replication, primates are high cost, there is limited macaque availability (especially females), and results are complicated by variations in host susceptibility because the animals are outbred and require larger group sizes to achieve statistically meaningful results. An alternative model for microbicide testing is the BLT mouse model [35–44]. Humanized BLT mice are generated by implanting human fetal liver and thymus tissues under the kidney capsule of an immunodeficient NOD scid gamma (NSG = NOD.Cg-*Prkdc*^*scid*^ *Il2rg*^*tm1Wjl*^/SzJ) mouse, followed by an administration of autologous human fetal liver CD34+ cells (human hematopoietic stem cells (HSC)). In BLT mice, T cell education occurs in the human thymic tissue, resulting in complete systemic reconstitution of all major human hematopoietic lineages including T, B, monocyte/macrophage, dendritic, and natural killer cells. Most importantly, the extensive systemic and genital mucosal reconstitution with human lymphoid cells renders female humanized BLT mice susceptible to both vaginal and rectal HIV infection [36–37]. This model is less expensive, less variable across study groups (production of sufficient numbers of mice from a single tissue donor), and better able to create group sizes that support stronger statistical comparisons.

The humanized mouse model has supported investigation of the effectiveness of antiretroviral (ARV) drugs dosed systemically [45] and topically [46–48] in preventing HIV. These studies have focused primarily on demonstrating that ARV drugs can prevent infection in humanized mice and validating the humanized mouse models for evaluating ARV candidates. Here we report a series of dose-response studies that allow determination of the half maximal effective concentrations (EC_50_) for HIV infection in BLT mice following topical application of TDF and FTC. These results are applied to an empirical HIV infection model [49], with the goal to identify an optimal combination of topical ARVs to confer protection against rectal or vaginal HIV challenge.

## Materials and methods

### Generation of humanized BLT mice

Humanized BLT mice were generated as described previously [44, 50–52], by implanting 1-mm^3^ pieces of human fetal liver and thymus tissues (Advanced Bioscience Resources) under the kidney capsule in 6 to 8-week-old female NSG mice (Jackson Laboratories) bred at The Scripps Research Institute (TSRI). Each cohort was produced with tissues from a single donor. CD34+ HSPC were purified from autologous fetal liver tissue, isolated by magnetic bead selection for CD34+ cells (Miltenyi), phenotyped cytometrically [44, 50–52], and cryopreserved until injection (200,000–350,000 CD34+ cells) into mice 3 weeks after Thy/Liv implantation. Human reconstitution in peripheral blood was verified by flow cytometry as described previously [44, 50–52]. Mice were maintained at the Department of Animal Resources (DAR) at TSRI in accordance with protocols approved by the TSRI Institutional Animal Care and Use Committee (Permit Number: 13–0001). This study was carried out in strict accordance with the recommendations in the Guide for the Care and Use of Laboratory Animals of the National Institutes of Health. All surgery was performed under sodium pentobarbital anesthesia, and all efforts were made to minimize suffering. The method of sacrifice used for the experimental mice is cervical dislocation. A power calculation was used to determine the sample size (number of mice/group).

### Vaginal and rectal exposure of humanized BLT mice to HIV

**Fig 1:**
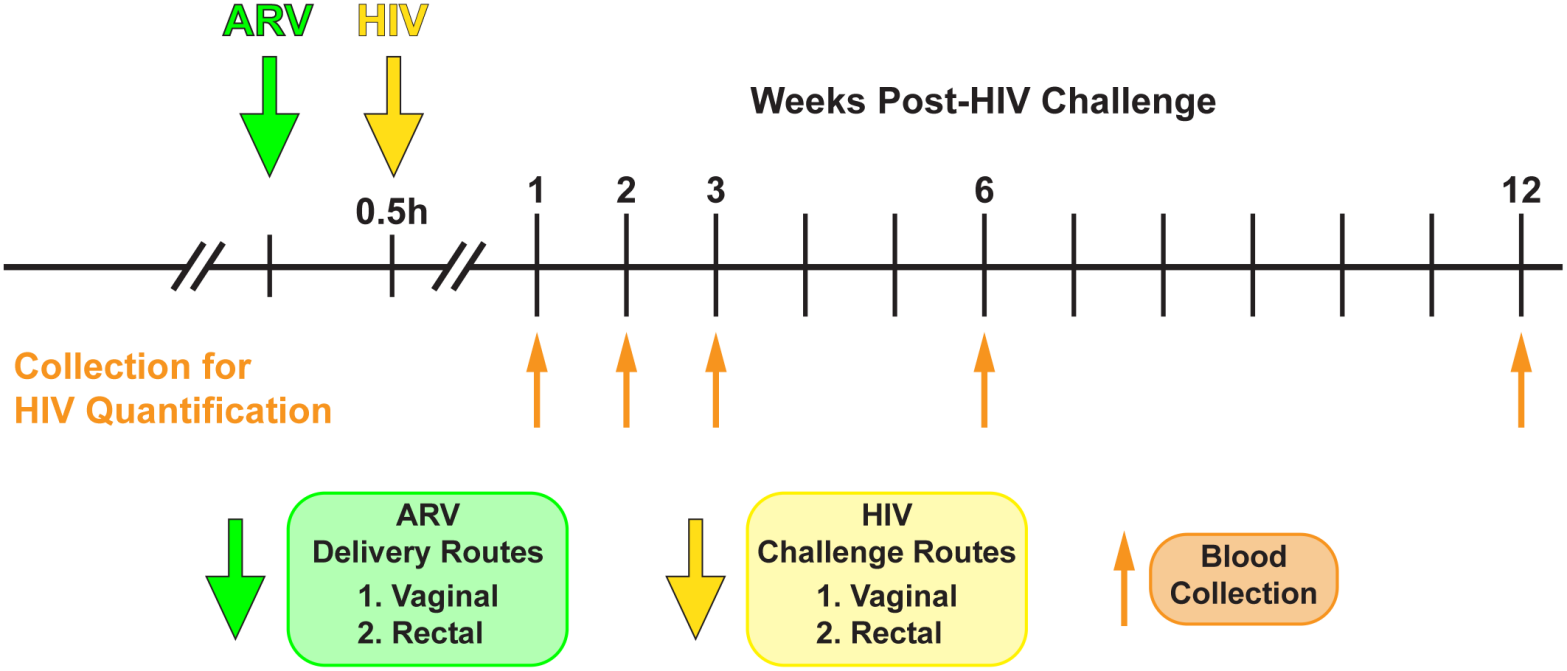
Experimental design. Humanized BLT mice were used to determine the efficacy of topically applied TDF and FTC to prevent both vaginal and rectal HIV transmission. Vaginal and rectal HIV exposures (yellow arrow) were conducted within 30 min (typically 10–15 min) following TDF and FTC application. Peripheral blood samples were collected at the indicated times (upward orange arrows) and viral load quantified by real-time PCR.

Three sets of dose-ranging infection studies were performed. The first used a broad dose range of single ARV (TDF: 8, 80, 800, 8,000 and 80,000 nM; FTC: 1, 10, 100, 1,000 and 10,000 nM) to map out the ideal dose range for EC_50_ determination. The second used a narrowed range based on results from the first study set (TDF: 1000–16,000 nM; FTC: 25–600 nM). The third set used a series of five combination doses of TDF and FTC in a 50:1 ratio (TDF + FTC: 2 μM + 0.04 μM, 4 μM + 0.08 μM, 8 μM + 0.15 μM, 16 μM + 0.3 μM, 32 μM + 0.6 μM). Each set of concentrations was used for vaginal and rectal challenges following the experimental design outlined in Fig 1. Stocks of HIV JR-CSF were prepared as previously described [44, 50] and standardized by p24 ELISA. Prior to inoculation, mice were anesthetized with isoflurane. Aliquots (5 μL) of TDF and/or FTC (Selleck Chemicals LLC) solutions in PBS were applied vaginally or rectally. Drugs were applied at the indicated concentration through a pipet tip into mice that had been anesthetized for the procedure. The rear half of the mouse was allowed to remain elevated during the procedure to reduce chance of back flow from the vaginal cavity during the recovery. Ten to fifteen minutes post-drug application, mice were vaginally challenged with HIV and infection and monitored by quantifying viral RNA by PCR viral load in peripheral blood at weeks 1, 2, 3, 6 and 12. The atraumatic vaginal and rectal HIV challenges were conducted as previously described [36–38, 40, 51] using a total volume of 5 μL (200 ng of p24).

### Pharmacokinetics studies

The tissue concentrations obtained following topical TDF and FTC dosing were evaluated in separate pharmacokinetics (PK) studies for vaginal and rectal dosing. Mice received vaginally or rectally applied TDF and FTC (TDF: 4, 8, and 32 μM; FTC: 0.08, 0.6, and 1 μM) following an identical dosing procedure to the infection studies, but eliminating the HIV inoculation step. At each time point (2h, 8h, 24h), three mice were sacrificed and the entire vagina, rectum and colon were removed at necropsy and flash-frozen in liquid N_2_ for analysis of drug concentrations.

### Analysis of HIV infection of humanized BLT mice

Infection of BLT mice was analyzed by quantifying HIV RNA levels in peripheral blood (plasma) using one-step reverse transcriptase quantitative real-time PCR (ABI custom TaqMan Assays-by-Design) according to the manufacturer’s instructions. Primers were 5-CATGTTTTCAGCATTATCAGAAGGA-3 and 5-TGCTTGATGTCCCCCCACT-3, and MGB-probe 5-FAM-CCACCCCACAAGATTTAAACACCATGCTAA-Q-3, where FAM is 6-carboxyfluorescein [36–38, 40, 51]; The assay sensitivity was of 300–500 400 RNA copies per mL.

### Bioanalysis of *in vivo* samples

Vaginal, rectal and colonic tissues were collected at predetermined time points following either vaginal or rectal dosing, and concentrations of TFV, TFV-DP, and FTC were determined via previously described liquid chromatographic-tandem mass spectrometric (LC-MS/MS) assays [53–54]. All assays were developed and validated following the Food and Drug Administration Guidance for Industry, Bioanalytical Method Validation recommendations and met all acceptability criteria. Isotopically labeled internal standards were used for all compounds and the determination of drug concentrations in all specimen sources. The lower limits of quantification for these methods were as follows: vaginal tissue homogenate, TFV (0.05 ng/sample), TFV-DP (50 fmol/sample), FTC (0.25 ng/sample). Tissue samples were ultimately reported as ng/mg or fmol/mg, respectively, following normalization to tissue mass for each sample. Testing was performed at the Johns Hopkins University School of Medicine Clinical Pharmacology Analytical Laboratory (Baltimore, MD, USA).

### Data analysis

Data were analyzed using GraphPad Prism (version 7.00, GraphPad Software, Inc., La Jolla, CA). For pharmacokinetic analyses, post-dose analyte concentrations below the corresponding LLOQs (*C*_*LLQ*_) were treated as:

$$C_{LLQ}=\frac{\text{LLOQ of assay}}{2\times \left(\text{median sample mass}\right)}$$ (1)

Analytic simulations of dose-response curves using the median-effect principle and mass-action law, and its combination index theorem [55] were carried out using CompuSyn [56].

## Results

### Protection by topical administration of TDF and FTC against both vaginal and rectal HIV challenge in humanized BLT mice

The degree of humanization of the BLT mice vas verified at 20 weeks of age (10 weeks post-CD34+ HSPC injection) prior to each challenge study by collecting peripheral blood and analyzing it by FACS for percentages of human CD45+ cells and human CD45+ CD4+ CD3+ cells. This data are shown in S1, S2 and S3 Figs (Supporting information), respectively, for each infectivity study. Mice that did not exhibit sufficient percentages of human cells (<65% of CD45+ cells and <70% of CD45+ CD4+ CD3+ cells) were not used in infection studies. After confirming the reconstitution of mice with human cells, a series of infection studies over a broad dose range were conducted to determine the drug concentrations applied vaginally or rectally that offer protection against rectal or vaginal HIV challenge.

Vaginally applied TDF offered no protection against a vaginal HIV challenge at 8, 80 and 800 nM, 66.7% protection at 8,000 nM and 100% at 80,000 nM, while FTC offered no protection at 1 and 10 nM, 33.3% protection at 100 nM and 100% at 1,000 and 10,000 nM (Table 1, S1 Fig). Infection of humanized mice remained steady between weeks 1 and 12 following vaginal challenge in untreated animals (S1 Fig). Rectally applied TDF offered no protection against a rectal challenge at 8, 80 and 800 nM, 50% protection at 8,000 nM and 100% at 80,000 nM, while FTC offered no protection at 1 and 10 nM, 50% protection at 100 nM and 100% at 1,000 and 10,000 nM (Table 1, S1 Fig). Similar to the vaginal challenge, HIV replication after rectal challenge remained steady from week 1 to 12 in untreated mice (S1 Fig).

**Table 1:** Results of broad-range (Round 1) dose ranging infection study.

TDF Conc. (nM)	Dosing/infection Route		FTC Conc. (nM)	Dosing/infection Route	
	Vaginal	Rectal		Vaginal	Rectal
8	0% (0/6)	0% (0/6)	1	0% (0/6)	0% (0/6)
80	0% (0/6)	0% (0/6)	10	0% (0/6)	0% (0/6)
800	0% (0/6)	0% (0/6)	100	0% (0/6)	50% (3/6)
8,000	67% (4/6)	50% (3/6)	1,000	50% (3/6)	100% (6/6)
80,000	100% (6/6)	100% (6/6)	10,000	100% (6/6)	100% (6/6)

Increasing concentrations of TDF or FTC were applied vaginally or rectally to humanized BLT mice (group of 6). After 15 min, mice were vaginally or rectally challenged with HIV and infection analyzed over a period of 12 weeks. Data are expressed as % of protection.

### Determination of the 50% effective concentrations (EC_50_) of TDF and FTC providing protection against vaginal and rectal HIV challenges in BLT mice

**Fig 2:**
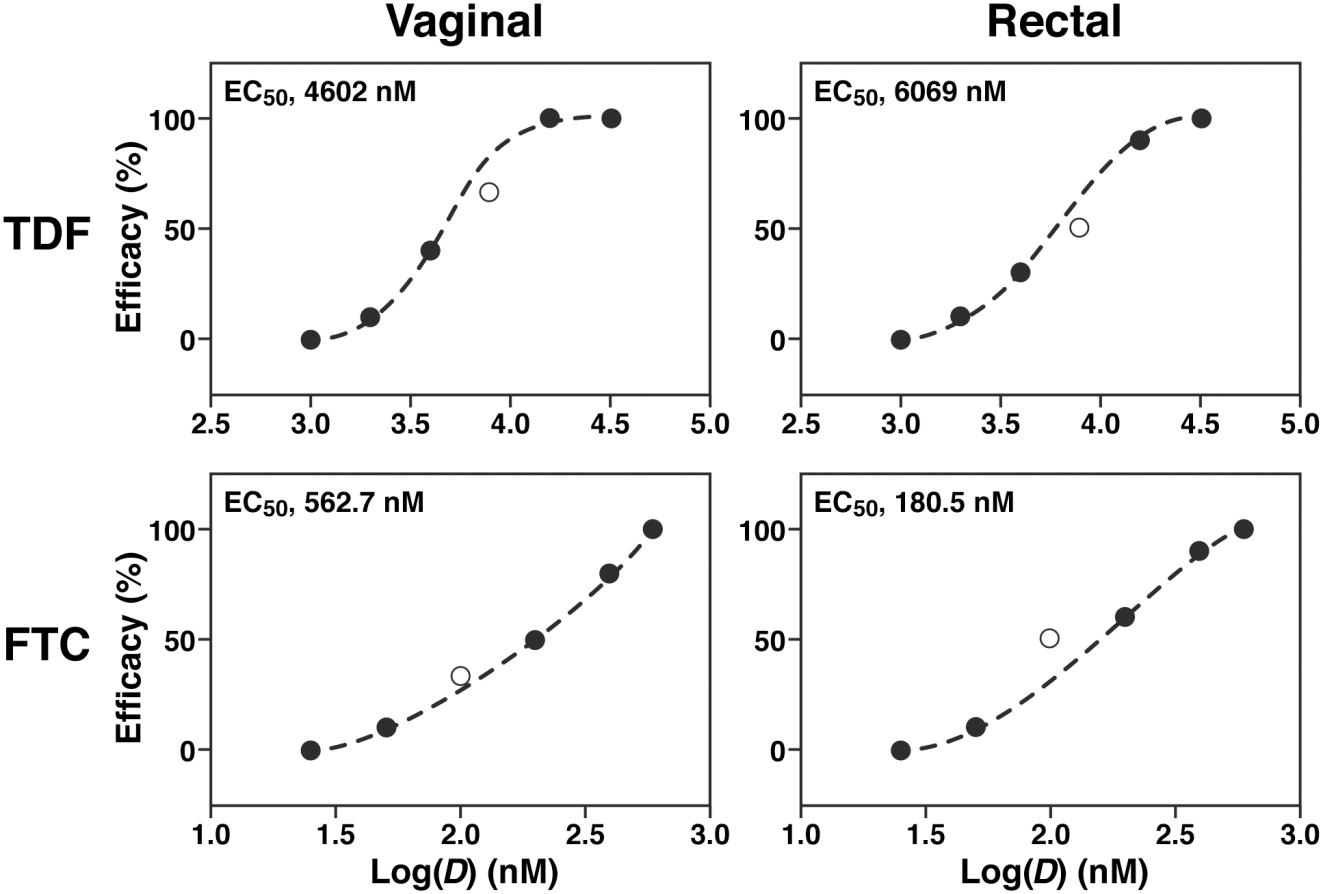
Dose-response curves for TDF and FTC single-drug infection studies. Plots of efficacy versus concentration of TDF or FTC dose applied prior to HIV challenge for Round 1 (broad concentration range, open circles) and Round 2 (narrow concentration range, filled circles). Dashed lines are fits to a sigmoidal dose-response (variable slope) model used to calculate EC_50_ of TDF and FTC providing protection against vaginal and rectal HIV challenges in humanized BLT mice.

Based on the efficacy data (Table 1), a narrower range of TDF and FTC doses were evaluated to map out the dose-response curve and allow calculation of EC_50_. In these second round studies, vaginally applied TDF offered no protection against a vaginal HIV challenge at 1,000 nM, 10% protection at 2,000 nM, 40% at 4,000 nM and 100% at 16,000 nM, while FTC offered no protection at 25 nM, 10% protection at 50 nM, 50% at 200 nM, 80% at 80 nM and 100% at 600 nM (Table 2, S2 Fig). For rectal challenge following rectal drug application, TDF provided no protection at 1,000 nM, 10% protection at 2,000 nM, 30% at 4,000 nM 90% at 16,000 nM and 100% at 32,000 nM, while FTC provided no protection at 25 nM, 10% protection at 50 nM, 60% at 200 nM, 90% at 400 nM and 100% at 600 nM (Table 2, S2 Fig). The 50% effective concentrations (EC_50_) of TDF and FTC that provided protection against vaginal and rectal HIV challenges in BLT mice were determined from the infection data using a sigmoidal dose-response (variable slope) model. The calculated EC_50_ values were: 4,600 nM for TDF and 563 nM for FTC applied vaginally, and 6070 nM for TDF and 181 nM for FTC applied rectally (Fig 2).

**Table 2:** Results of narrow-range (Round 2) dose ranging infection study for EC_50_ determination.

TDF Conc. (nM)	Dosing/infection Route		FTC Conc. (nM)	Dosing/infection Route	
	Vaginal	Rectal		Vaginal	Rectal
1000	0% (0/10)	0% (0/10)	25	0% (0/10)	0% (0/10)
2000	10% (1/10)	10% (1/10)	50	10% (1/10)	10% (1/10)
4000	40% (4/10)	30% (3/10)	200	50% (5/10)	60% (6/10)
16,000	100% (10/10)	90% (9/10)	400	80% (8/10)	90% (9/10)
32,000	100% (10/10)	100% (10/10)	600	100% (10/10)	100% (10/10)

Increasing concentrations of TDF or FTC were applied vaginally or rectally to humanized BLT mice (group of 6). After 15 min, mice were vaginally or rectally challenged with HIV and infection analyzed over a period of 12 weeks. Data are expressed as % of protection.

### TDF/FTC combination studies in humanized BLT mice

Based on the single-drug dose-response studies, an escalating-dose series of TDF + FTC combinations were evaluated for efficacy in the same BLT mouse infection model. When vaginally applied, the 2 μM TDF + 0.04 μM FTC combination provided no protection against a vaginal HIV challenge, 4 μM TDF + 0.08 μM FTC offered 50% protection, 8 μM TDF + 0.15 μM FTC produced 70% protection, 16 μM TDF + 0.3 μM FTC offered 70% protection, and 32 μM TDF + 0.6 μM FTC provided 100% protection (Table 3). Similarly, for rectal application, the 2 μM TDF + 0.04 μM FTC combination offered no protection against a rectal HIV challenge, the 4 μM TDF + 0.08 μM FTC combination provided 30% protection, 8 μM TDF + 0.15 μM FTC produced 60% protection, 16 μM TDF + 0.3 μM FTC offered 50% protection, and 32 μM TDF + 0.6 μM FTC provided 90% protection (Table 3).

**Table 3:** Results of TDF + FTC combination (Round 3) dose ranging infection study.

TDF Conc. (nM)	FTC Conc. (nM)	Dosing/infection Route	
		Vaginal	Rectal
2000	40	0% (0/10)	0% (0/10)
4000	80	50% (5/10)	30% (3/10)
8000	150	70% (7/10)	60% (6/10)
16,000	300	70% (7/10)	50% (510)
32,000	600	100% (10/10)	90% (9/10)

Increasing concentrations of TDF or FTC were applied vaginally or rectally to humanized BLT mice (group of 6). After 15 min, mice were vaginally or rectally challenged with HIV and infection analyzed over a period of 12 weeks. Data are expressed as % of protection.

### EC_50_ and median effect model calculations

**Fig 3:**
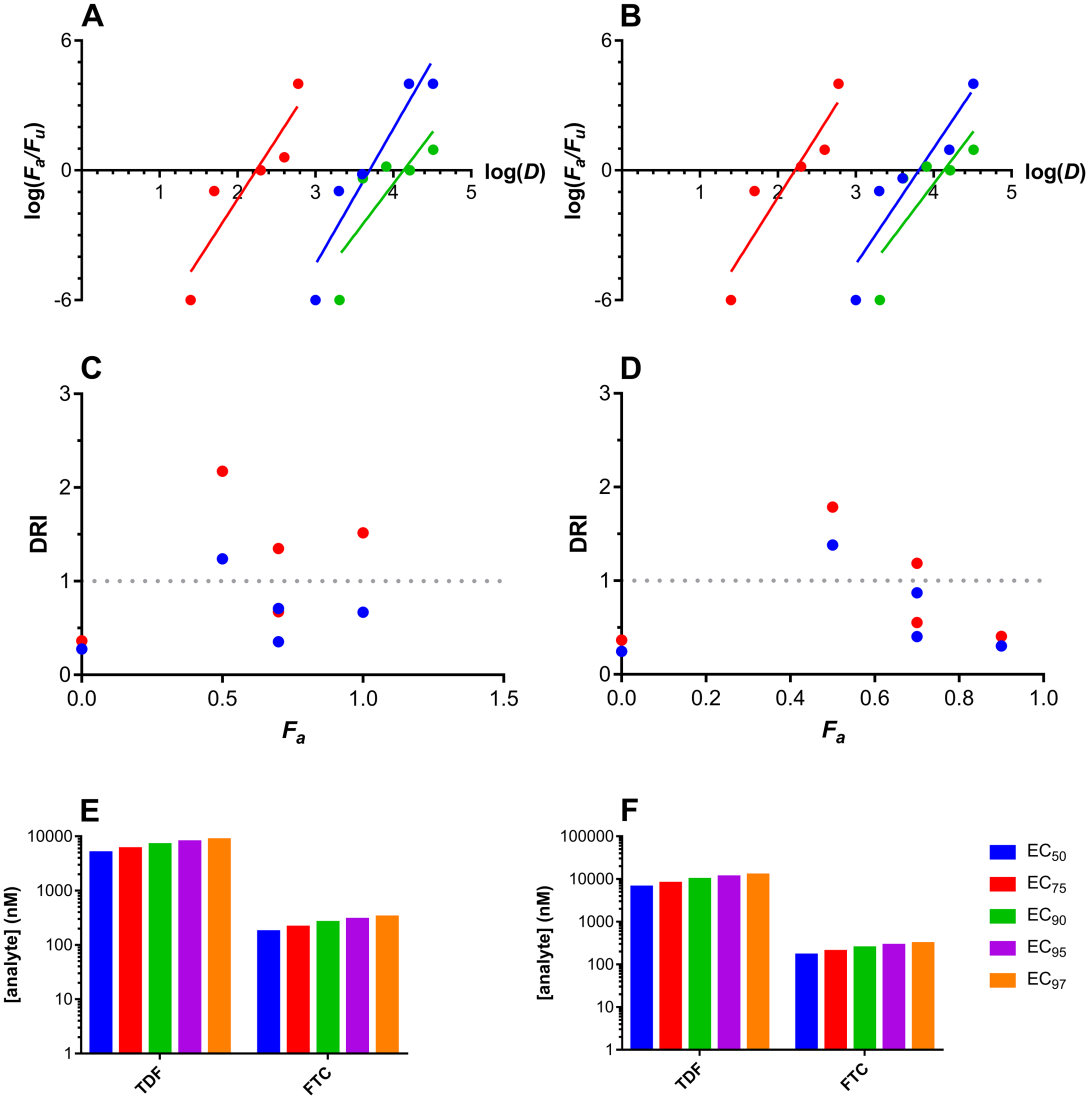
TFV and TFV-DP tissue concentration-time profiles following either vaginal or rectal dosing using 32 μM TDF. Median effect plots for (A) vaginal and (B) rectal drug dosing. *F*_*a*_, fraction affected; *F*_*u*_, fraction unaffected; *D*, dose (nM); blue circles, TDF; red circles, FTC; green circles, TDF-FTC combination. Dose response index (DRI) plots for (C) vaginal and (D) rectal dosing of both drugs in the TDF-FTC combination. *F*_*a*_, fraction affected; blue circles, TDF; red circles, FTC. The DRI of 1 shown in as a broken line represents no dose reduction relative to the drugs evaluated individually. Predicted EC_50_-EC_97_ values for the TDF-FTC combination for (E) vaginal and (F) rectal drug dosing.

Analyzing the above dose-response relationships using the median-effect model based on mass action [55] allowed important parameters in addition to EC_50_ values to be calculated (Fig 3). The slope parameter, *m*, is analogous to the Hill coefficient and describes the sigmoidicity of the dose-effect curve. For single drug administration, the following slopes (*m*) were calculated: TDF, vaginal, 6.3 ± 1.2, rectal, 5.4 ± 1.3; FTC, vaginal, 5.6 ± 1.5, rectal, 5.7 ± 1.4. The combination index (*CI*) allows potential synergism, summation, or antagonism effects resulting from drug combinations to be quantified empirically. For the TDF-FTC combination, *CI* values > 0 were obtained for vaginal and rectal administration and HIV challenge, suggesting that the two ARV agents were slightly to moderately antagonistic. A dose-reduction index (DRI) analysis of the drug combination did not identify a consistent effect across multiple effect levels.

### Pharmacokinetics of topical TDF

**Fig 4:**
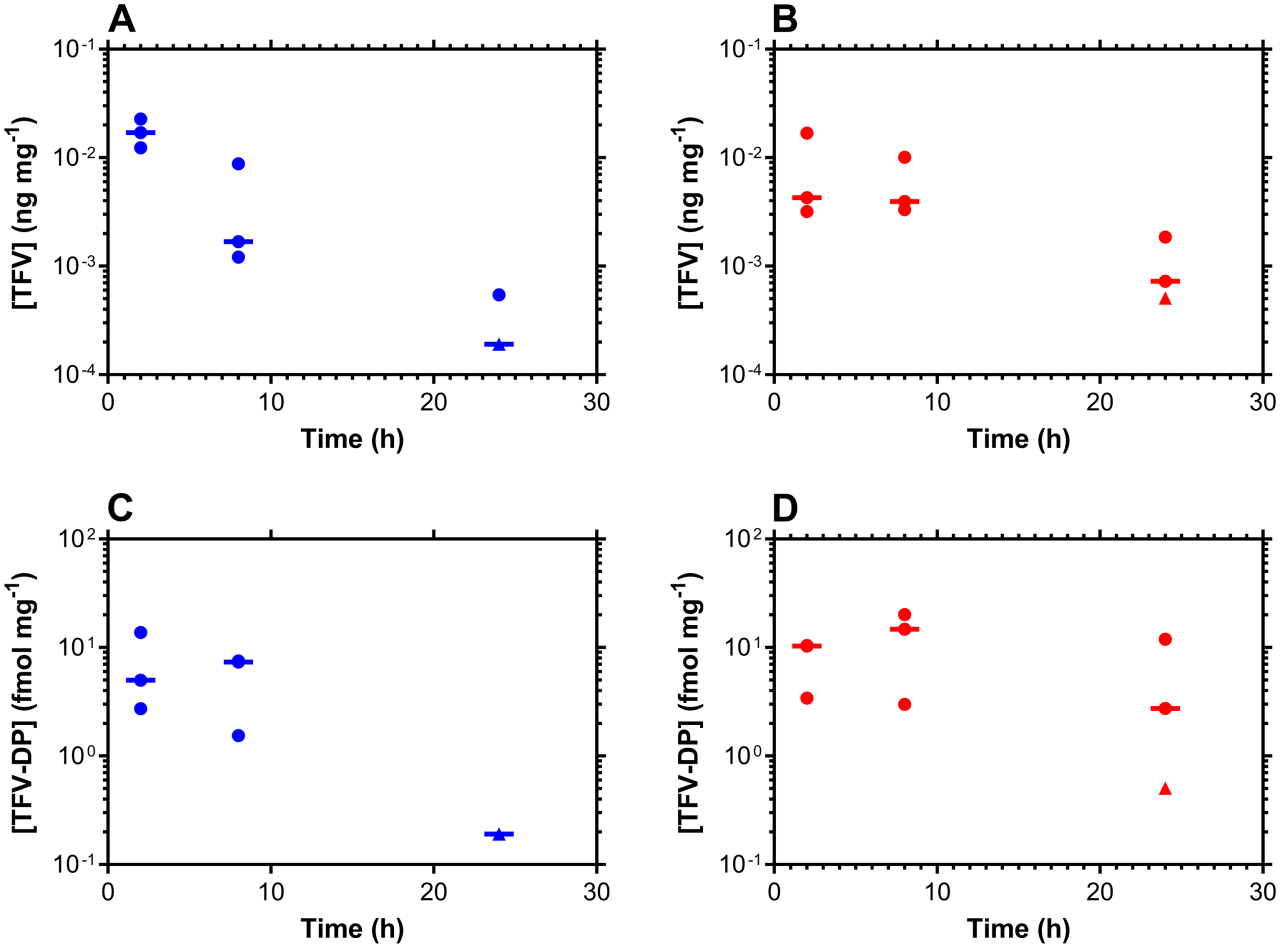
Dose-response relationships analyzed using the median effect model. Horizontal lines represent group medians; every circular datum represents an individual sample from one of the animals (n = 3); triangles depict samples that were below the lower limit of quantitation (BLQ) of the analytical method, and values were calculated as follows: [(assay BLQ)/2]/(median tissue mass). TFV tissue concentrations following vaginal (A, blue) and rectal (B, red) dosing; TFV-DP tissue concentrations following vaginal (C, blue) and rectal (D, red) dosing.

Vaginal, rectal, and colon tissue collected at predetermined time points following either vaginal or rectal dosing were analyzed for TFV, TFV-DP, the active metabolite of TFV, and FTC. Vaginal, rectal, and colonic tissue FTC concentrations in all groups (0.08, 0.6, and 1 μM single vaginal or rectal dose) were below the limit of quantification of the assay, even though these concentrations provided 80–100% protection from HIV transmission in the corresponding anatomic compartments. The median lower limit of quantification for FTC concentrations in vaginal and rectal tissues are 1.9 and 5.1 pg/mg, respectively. Vaginal, rectal, and colonic tissue TFV and TFV-DP concentrations also were below the lower limit of quantification of the assay in most groups (4 and 8 μM single vaginal or rectal dose), but were quantifiable in in most samples in the 32 μM dosing groups (Fig 4). In colonic tissue, the concentrations of TFV could only be quantified at 2 h (median, 4.1 pg/mg) and 8 h (median, 14 pg/mg), while TFV-DP concentrations were only quantifiable at 8 h (3.6 fmol/mg) post-dose. Based on aforementioned assay LLOQs, and the dosages applied, a full PK-PD analysis was not performed. However, we were able to measure the vaginal/rectal tissue TFV and TFV-DP concentration-time profile at the highest dose.

## Discussion

It is now well established that PrEP with ARV drugs delivered orally [11–16] or topically [2–10, 17–24] can significantly reduce HIV infection in individuals who are adherent to the drug regimen. Although there are more than 25 FDA approved small-molecule anti-HIV drugs from six mechanistic classes, all but one of the PrEP approaches demonstrating clinical efficacy are based on TFV, or its TDF prodrug, alone or in combination with FTC. Mechanistically, both of these drugs are nucleoside reverse transcriptase inhibitors (NRTIs), and therefore act on replicating virus in an early, focal infection. Similar to successful approaches in HIV treatment, it is likely that ARV combinations will lead to more effective PrEP with decreased resistance development compared to single-drug approaches [57]. The rigorous determination of optimal ARV combinations for HIV prevention, however, has been limited by the lack of models that are cost-effective, reproduce the complexities of mucosal HIV infection, and allow the statistical power to observe differences between different ARV combinations and doses. The use of SIV and SHIV infection models in non-human primate (NHP) studies has been the primary approach to evaluate PrEP efficacy pre-clinically [58]. The NHP methods suffer from two major drawbacks: the high cost and limited availability of animals limits the power of NHP studies, and differences between HIV and SIV or SHIV can confound translation of NHP study results to clinical efficacy [46].

The BLT humanized mouse model is relatively low-cost and has been shown to faithfully replicate the human female reproductive tract with regards to immune cell populations (42). Humanized BLT mice are susceptible to a high rate of rectal and vaginal transmission of HIV across an intact epithelium, indicating the potential to study sustained release of drugs from IVRs and to analyze their protection efficacy. To date, most humanized mouse efficacy studies of ARV-based HIV PrEP have examined dosing regimens targeting complete protection (100% efficacy) in an approach similar to that commonly used in NHP studies. The studies described here demonstrate that the humanized mouse model can be utilized in dose-ranging HIV infection studies that allow the determination of EC_50_ values and other dose-response parameters related to preventing genital HIV transmission. A major advantage of the humanized mouse model is the possibility of using high numbers of mice per treatment (n = 10) and a large number of treatment groups (5–6) in a single set of experiments in order to reach a sufficient statistical power to utilize these dose-response methods in an *in vivo* model. These studies have typically been limited to *in vitro* models, and to our knowledge, this is the first study to thoroughly evaluate the dose-response behavior of HIV prevention modalities *in vivo*.

To validate this approach for PrEP with ARV agents applied topically, we first evaluated the efficacy of two well-characterized NRTIs, TDF and FTC, at preventing vaginal and rectal HIV transmission using the humanized BLT mouse model. Previous studies showed that BLT mice are highly susceptible to both vaginal and rectal HIV infections [36–38, 40, 51–52]. An initial (Round 1) dose-ranging infection study demonstrated that topical administration of unformulated TDF or FTC can offer complete protection against either a vaginal or rectal HIV challenge. These results provided guidance for narrowing the dose range to be used in the Round 2 studies to accurately determine the 50% effective concentrations (EC_50_) of TDF and FTC providing protection against vaginal and rectal HIV challenges when administered vaginally or rectally as unformulated, single-drug solutions.

The TDF and FTC dose ranges for the Round 2 infection studies were chosen so that ideally 2–3 intermediate efficacy values (10–90%) would be obtained, allowing accurate fits (*R*^*2*^ = 0.9993 and 0.9997 for vaginal and rectal administration, respectively) of the data to a sigmoidal dose-response model (Fig 2). The fact that the efficacy values from Round 1 studies (Fig 2, open circles) fall approximately on the dose-response curve calculated from the Round 2 data (Fig 2, filled circles) indicates the robustness and reproducibility of the BLT mouse infectivity model, a significant result. The EC_50_ values calculated from the dose-response data are consistent with, but at the high end of the range of IC_50_ values reported for TDF and FTC *in vitro*. For measurements in lymphoblastoid cell lines, the MAGI-CCR5 cell line, and peripheral blood mononuclear cells, IC_50_ values in the range 40–8500 nM for TDF and 1.3–640 nM for FTC were obtained [59]. Similar values in the range 500–2250 nM for TDF and 7–75 nM for FTC were obtained in cell culture with HIV-1 clades A, B, C, D, E, F, and G [59]. An IC_50_ of 90 nM was measured *in vitro* for inhibition of HIV infection by TDF in JT-CCR5 cells inoculated with lab-adapted HIV-1_BaL_ [60].

Because reported *in vivo* HIV infection studies have typically employed dosing levels aimed at achieving complete protection (*i*.*e*. doses significantly above *in vitro* IC_50_ or IC_90_), there is limited *in vivo* data to compare efficacious cervicovaginal fluid (CVF) or tissue concentrations in other model systems with the EC_50_ values obtained from the BLT mouse studies. A vaginal ring delivering TDF and FTC protected 6/6 animals in a pigtail macaque low-dose repeat challenge SHIV infection model [61]. Median undiluted CVF levels of TDF (7.24 × 10^4^ nM) and FTC (4.45 × 10^6^ nM) in the macaque study were 13× higher for TDF and 7900× higher for FTC than the EC_50_ vaginal dose (TDF: 4600 nM; FTC: 560 nM). For a TDF-FTC gel in a similar macaque model, TDF and FTC doses protecting 6/6 macaques were significantly higher than in the vaginal ring study (1.57 × 10^7^ nM for TDF and 2.02 × 10^8^ nM for FTC) [62]. The TDF EC_50_ levels in BLT mice are similar to the CVF concentrations obtained from efficacious PrEP oral dosing (8.47 × 10^3^ nM) [63] and to the CVF level associated with significant efficacy against HIV infection in the CAPRISA 004 trial of a pericoital 1% TFV gel (1.57 × 10^3^ nM) [64]. However, it is important to emphasize that the EC_50_ values calculated in the present study were determined based on the concentration of drug directly administered to the vagina or rectum, not based on the concentrations present in the genital fluid or in target tissues. Due to the short time between topical administration of drug and the HIV exposure (15–30 min), distribution of the drug in the target tissues is different when compared to systemic drug administration. The type of drug formulation can have a significant impact on bioavailability, including drug mucosal penetration, the ability to target draining lymph nodes, and cellular uptake kinetics (e.g. solution in a gel vs. nanoparticles). We also indicate in this study that we exclusively formulated in PBS, allowing comparison of different drugs and drug combinations; however, The EC90, and the other median-effects may vary with other type of formulations such as intravaginal rings (IVRs).

The single-drug and combination TDF and FTC studies demonstrate that the median-effect model can be applied to dose-response infectivity results in BLT mice to quantify efficacy with single agents and characterize the effectiveness of ARV combinations. The *m* value is the slope of the log-log relationship described by the median-effects plots (Fig 3A and 3B), providing a quantitative measure of sigmoidicity (i.e., kinetic order) [65]. The high regression coefficients (*r*, 0.91–0.95) support the applicability of the model to the analysis. Shen *et al*. used an *ex vivo* HIV model to show that ARV agents have a characteristic slope, ranging from 1 for NRTIs to 1.8–4.5 for protease inhibitors [66]. The slopes (*m*) of the TDF (6.3 and 5.4 for vaginal and rectal dosing, respectively) and FTC (5.6 and 5.7 for vaginal and rectal dosing, respectively) dose-response curves *in vivo* obtained here were much higher than the *ex vivo* values reported by Shen and colleagues [66]. These observations need to be taken into account when predicting the drug concentrations required for full HIV prevention efficacy (i.e., EC_100_) based on *ex vivo* and *in vivo* EC_50_ values. When *m* > 1, the dose-response curves are steep and a small increase in drug concentration may have large effects. The higher the *m*-value, the lower the EC_100_/EC_50_ concentration ratio.

### Analysis of TDF-FTC combinations

Jilek *et al*. used an *in vitro* single-round infectivity assay and several empirical models to study the effect of numerous ARV drug combinations on HIV replication in an effort to predict drug efficacy in ARV therapy [67]. The researchers found that the TDF-FTC combination had an intermediate inhibitory effect attributed to the fact that both agents target reverse transcriptase. The robust BLT mouse model of topical HIV prevention described here allowed the dose-response behavior of single and combination ARV drugs to analyzed using the median effect model [65]. To our knowledge, ours is the first account to empirically investigate the effect of drug combinations on the prevention of rectal and vaginal HIV infection *in vivo*. We found that in both anatomic compartments, the TDF-FTC combination exhibited slight-moderate antagonism (CI > 1) [68] at *F*_*a*_ levels between 0.3 and 1.0 (median CI, 2.2 vaginal dosing; 3.1, rectal dosing), as has been observed in the case of two mutually non-exclusive enzyme inhibitors [65]. The dose-reduction index (DRI) describes the reduction or increase in dose observed for a combination compared to the individual ARV drugs to achieve the same effect, with a DRI > 1 indicating a reduction in dose is observed for the combination. The lack of a consistent DRI across effects levels (Fig 3C and 3D) observed here suggests that the antagonism does not have a systematic impact on the drug doses required for efficacy (Fig 3E and 3F), which take into account the observed antagonistic effects of the two agents. Note that we are aware that we cannot extrapolate from ARV for HIV treatment to ARV for prevention. ARVs inhibit HIV replication when used for treatment; however, there is no HIV replication during prevention of HIV transmission (unless the prevention fails). Except if there is a putative slight HIV replication in residual vaginal macrophages in the early stages of colonization, resistance development should not occur during ARV vaginal or rectal application (unless the prevention fails).

To be suitable for HIV transmission studies after intravaginal or rectal challenge, BLT mice must be anatomically similar to humans at the proposed site of HIV entry, and with a sufficient reconstitution with human hematopoietic cells to support HIV transmission. A previous study nicely described these similarities [69]. The anatomy of the mouse female genital tract is very similar to the human despite its smaller size and the presence of a double uterus. The uterine horns merge caudally to form an undivided corpus uteri and a single cervical canal, which projects into the upper vagina. The murine vagina and endocervix are covered with a stratified squamous epithelium, whereas the endocervix and uterus consist of a simple columnar epithelium. The physical barrier HIV would encounter is, therefore, similar to that in humans. After reconstitution with human hematopoietic cells, the vagina, ectocervix, endocervix, and uterus of the BLT mice are repopulated with human T cells, macrophages, and DCs. Human T cells are present within the epithelial layer and the lamina propria, and macrophages and DCs are present in the lamina propria throughout the genital tract, reproducing the distribution of these cells in human genital tract.

In summary, the studies reported here represent the first demonstration that the BLT mouse model is a valuable tool for determination of EC_50_ values for the inhibition of vaginal or rectal HIV transmission by ARV drugs applied topically. This approach should allow the preclinical determination of optimized drug combinations for the prevention of HIV transmission. Based on the results of the single-drug and combination studies presented here, the BLT mouse infection model will next be used to evaluate a third ARV with a different mechanism of action in combination with TDF and FTC to test the hypothesis that a three-drug combination will diminish the required ARV concentrations, while preserving protection. Our ultimate goal is to determine the optimal ARV combination in murine IVR formulations and evaluate them for sustained and extended protection of humanized mice against vaginal and rectal HIV challenges as part of the pre-clinical development path of a combination pod-IVR nBP [70–72].

## Supporting information

S1 Fig Results of broad-range (round 1) dose ranging infection study. On the left of the panels the numbers of mice per treatment are indicated (n = 6) as well as the percentages of human CD45+ and CD45+ CD4+ CD3+ cells. Indicated in the middle of the panels are the numbers of 10^5^ copies per mL of plasma collected at weeks 1, 2, 3, 6 and 12. Indicated on the right of the panels are the concentrations of TDF and FTC applied vaginally (A) or rectally (B) 15 min prior to a vaginal (A) and rectal (B) HIV challenge.
(TIF)

S2 Fig Determination of EC_50_ of TDF and FTC providing protection against vaginal and rectal HIV challenges in BLT mice. On the left of the panels the numbers of mice per treatment are indicated (n = 10) as well as the percentages of human CD45+ and CD45+ CD4+ CD3+ cells. Indicated in the middle of the panels are the numbers of 10^5^ copies per mL of plasma collected at weeks 1, 2, 3, 6 and 12. On the right of the panels, the concentrations of TDF and FTC applied vaginally (A) and rectally (B) 15 min prior to a vaginal (A) and rectal (B) HIV challenge are indicated.
(TIF)

S3 Fig TDF/FTC combination studies in humanized BLT mice. Indicated on the left of the panels are the numbers of mice per treatment (n = 10) as well as the percentages of human CD45+ and CD45+ CD4+ CD3+ cells. In the middle of the panels the numbers of 10^5^ copies per mL of plasma collected at weeks 1, 2, 3, 6 and 12 are indicated. Indicated on the right of the panels are the concentrations of the combinations of TDF and FTC applied vaginally (A) and rectally (B) 15 min prior to a vaginal (A) and rectal (B) HIV challenge.
(TIF)
