# Supplementary figures and images for: Prevention of vaginal and rectal HIV transmission by antiretroviral combinations in humanized mice
Source: PLoS One. 2017 Sep 7;12(9):e0184303. doi: 10.1371/journal.pone.0184303 (PMC5589224; doi:10.1371/journal.pone.0184303)

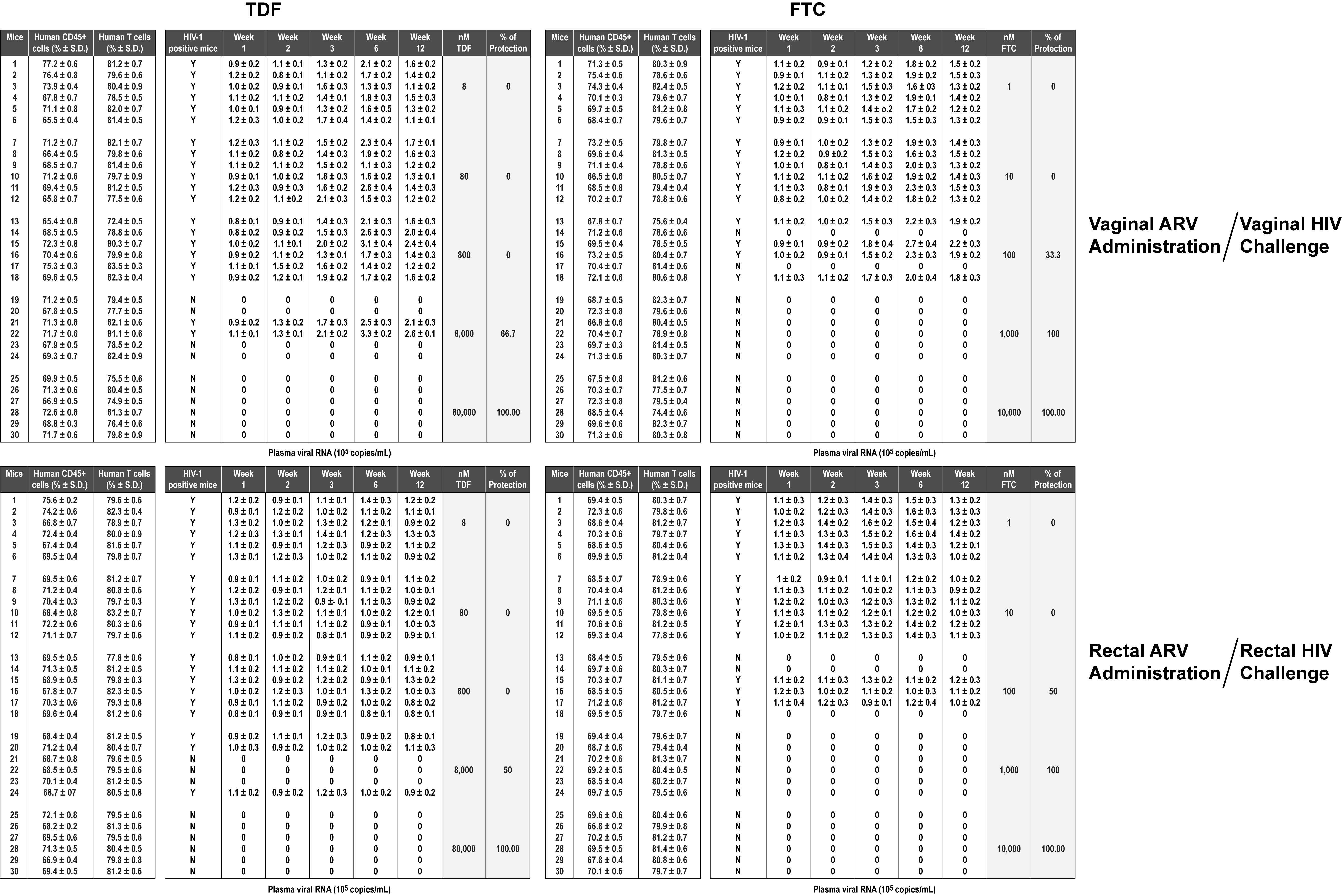

Supplement: S1 Fig — On the left of the panels the numbers of mice per treatment are indicated (n = 6) as well as the percentages of human CD45+ and CD45+ CD4+ CD3+ cells. Indicated in the middle of the panels are the numbers of 105 copies per mL of plasma collected at weeks 1, 2, 3, 6 and 12. Indicated on the right of the panels are the concentrations of TDF and FTC applied vaginally (A) or rectally (B) 15 min prior to a vaginal (A) and rectal (B) HIV challenge. (TIF) [file pone.0184303.s001.tif]

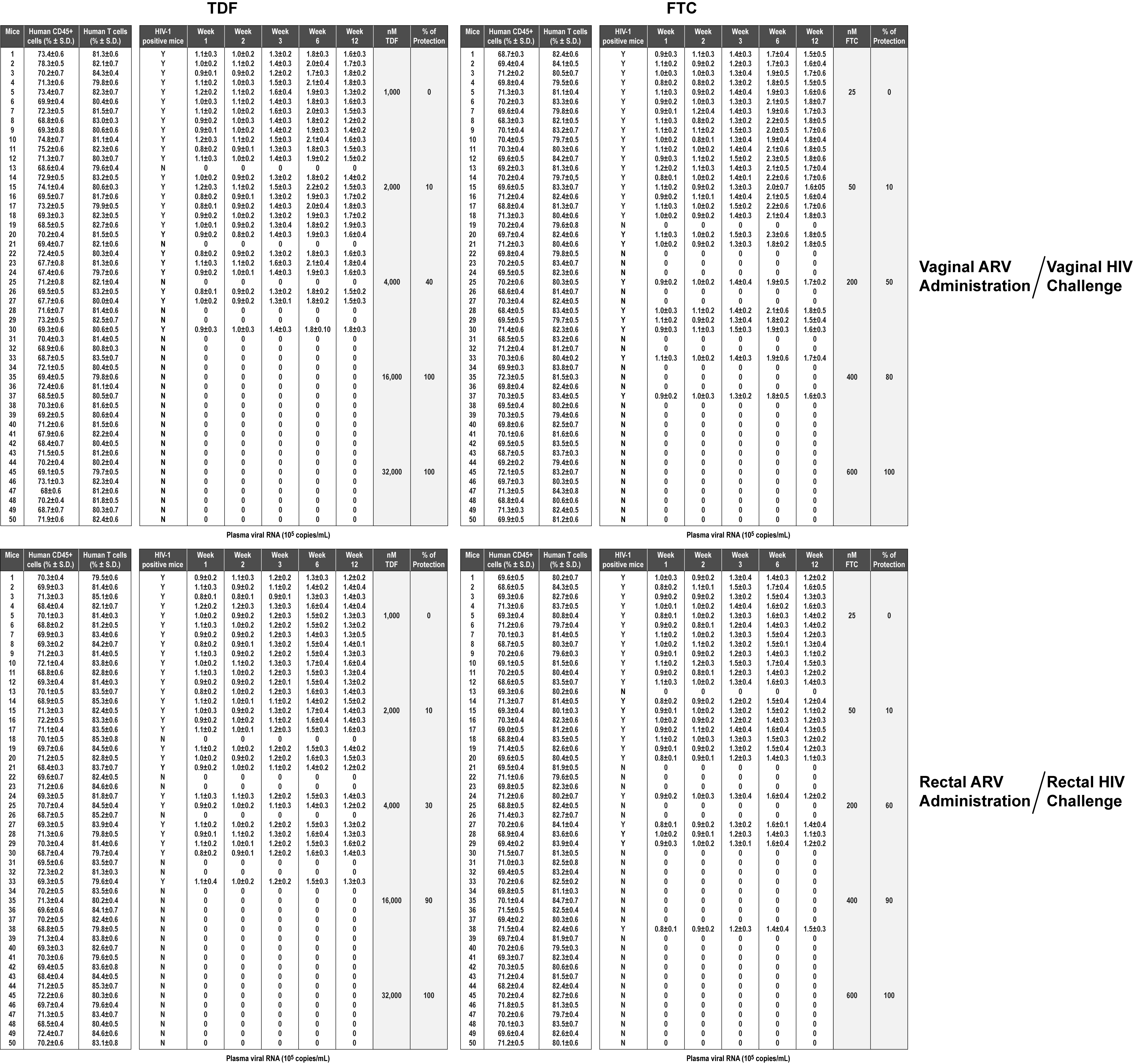

Supplement: S2 Fig — On the left of the panels the numbers of mice per treatment are indicated (n = 10) as well as the percentages of human CD45+ and CD45+ CD4+ CD3+ cells. Indicated in the middle of the panels are the numbers of 105 copies per mL of plasma collected at weeks 1, 2, 3, 6 and 12. On the right of the panels, the concentrations of TDF and FTC applied vaginally (A) and rectally (B) 15 min prior to a vaginal (A) and rectal (B) HIV challenge are indicated. (TIF) [file pone.0184303.s002.tif]

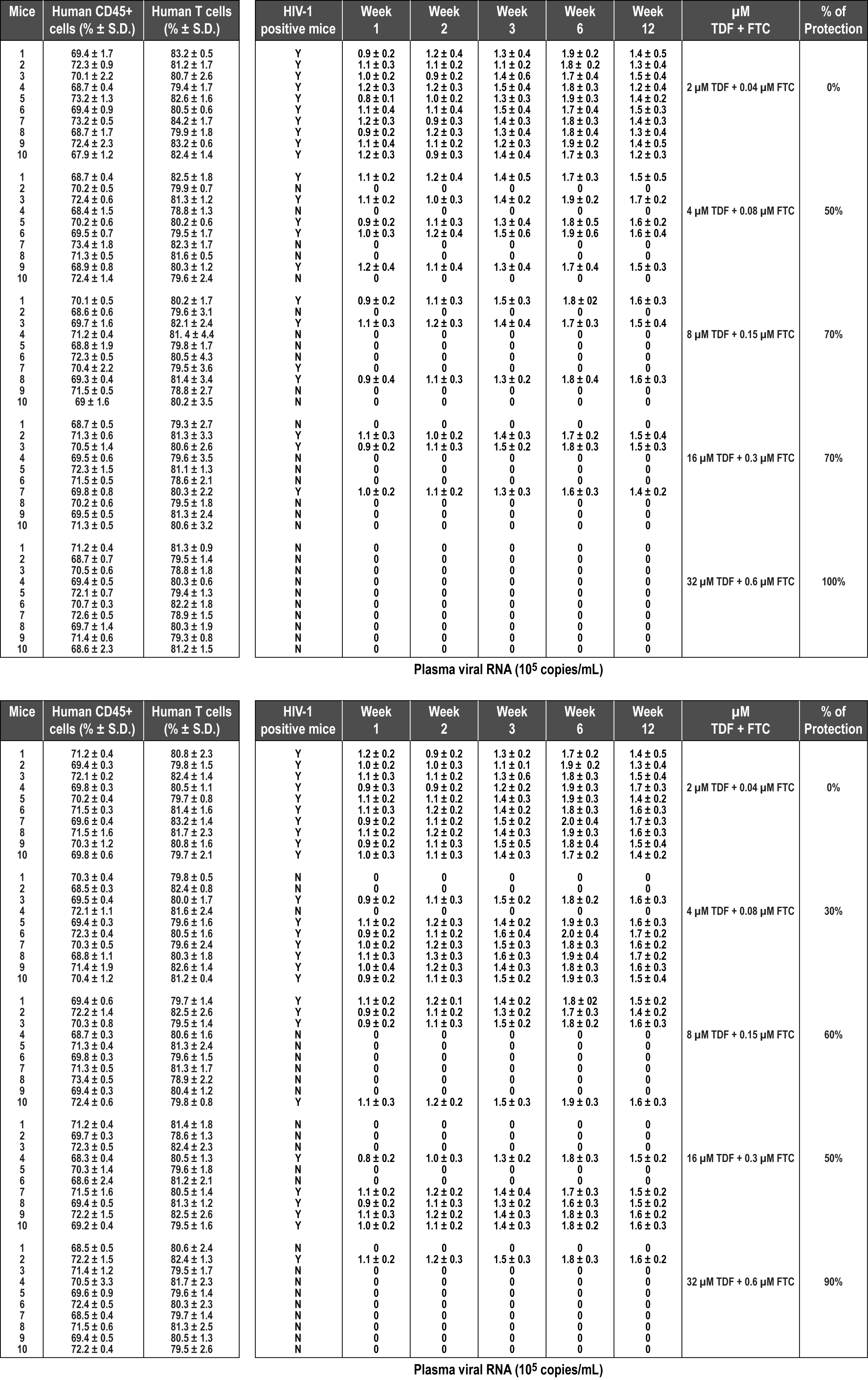

Supplement: S3 Fig — Indicated on the left of the panels are the numbers of mice per treatment (n = 10) as well as the percentages of human CD45+ and CD45+ CD4+ CD3+ cells. In the middle of the panels the numbers of 105 copies per mL of plasma collected at weeks 1, 2, 3, 6 and 12 are indicated. Indicated on the right of the panels are the concentrations of the combinations of TDF and FTC applied vaginally (A) and rectally (B) 15 min prior to a vaginal (A) and rectal (B) HIV challenge. (TIF) [file pone.0184303.s003.tif]
